# Correction: Brownian dynamics simulation of protofilament relaxation during rapid freezing

**DOI:** 10.1371/journal.pone.0253684

**Published:** 2021-06-17

**Authors:** Evgeniy V. Ulyanov, Dmitrii S. Vinogradov, J. Richard McIntosh, Nikita B. Gudimchuk

The affiliation for the fourth author is incorrect. Nikita B. Gudimchuk is not affiliated with #3 but with #2. The full affiliation for Nikita B. Gudimchuk is as follows:

**1** Department of Physics, Lomonosov Moscow State University, Moscow, Russia, **2** Center for Theoretical Problems of Physicochemical Pharmacology, Moscow, Russia, **4** Dmitry Rogachev National Medical Research Center of Pediatric Hematology, Oncology and Immunology, Moscow, Russia
