# Supplementary figures and images for: Brownian dynamics simulation of protofilament relaxation during rapid freezing
Source: PLoS One. 2021 Feb 12;16(2):e0247022. doi: 10.1371/journal.pone.0247022 (PMC7880439; doi:10.1371/journal.pone.0247022)

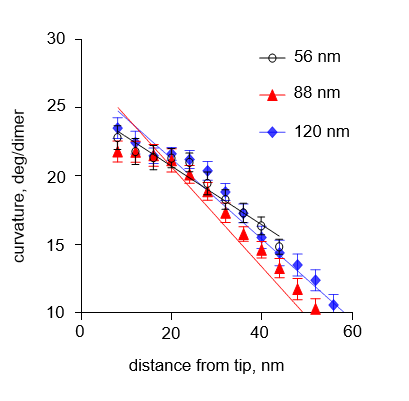

Supplement: S1 Fig — Data for 56, 88, and 120 nm-long filaments with persistence length about 2.1 μm, whose freezing was simulated with the cooling rate of 1.28·107 K/s. (TIF) [file pone.0247022.s001.tif]
